# Lower serum magnesium levels are associated with a higher risk of fractures and vascular calcifications in hemodialysis patients

**DOI:** 10.1093/ckj/sfae381

**Published:** 2024-11-28

**Authors:** Patrícia João Matias, Gonçalo Ávila, Diogo Domingos, Célia Gil, Aníbal Ferreira

**Affiliations:** Dialverca – Dialysis clinic, Forte da Casa, Portugal; NephroCare Carregado – Dialysis clinic, Carregado, Portugal; NOVA Medical School and Centro Clínico Académico de Lisboa, Lisboa, Portugal; Dialverca – Dialysis clinic, Forte da Casa, Portugal; Dialverca – Dialysis clinic, Forte da Casa, Portugal; Dialverca – Dialysis clinic, Forte da Casa, Portugal; NephroCare Carregado – Dialysis clinic, Carregado, Portugal; Dialverca – Dialysis clinic, Forte da Casa, Portugal; NephroCare Carregado – Dialysis clinic, Carregado, Portugal; NOVA Medical School and Centro Clínico Académico de Lisboa, Lisboa, Portugal

**Keywords:** bone fractures, hemodialysis, magnesium, secondary hyperparathyroidism, vascular calcifications

## Abstract

**Background:**

Magnesium (Mg) deficiency seems to be associated with altered bone metabolism and vascular calcifications (VC). This study aimed to evaluate the association between serum Mg levels and incident bone fragility fractures and VC in a cohort of prevalent hemodialysis (HD) patients.

**Methods:**

We performed a retrospective study of 206 patients, with a mean age of 68.3 ± 13.1 years; 121 (59%) were male, and the median follow-up time was 58 months.

**Results:**

Thirty-seven episodes of fragility fractures were identified with a median HD vintage of 42 months—an incidence rate of 29 per 1000 person-years. Patients with fractures showed lower Mg levels compared with those without fractures (*P* < .001) and more VC (*P* = .01). In a Cox regression analysis, time to fragility fracture was independently associated with serum Mg <2.2 mg/dL (*P* < .001), in a model adjusted to age, female gender, HD vintage, diabetes mellitus, body mass index, albumin, parathyroid hormone, active vitamin D therapy and the presence of VC. Patients with Mg serum levels <2.2 mg/dL had a 1.32-fold higher risk of fragility fractures (*P* < .001).

**Conclusions:**

This study showed that the incidence of bone fragility fractures in HD patients is high and is significantly associated with lower Mg levels and with the presence of more VC.

## Graphical Abstract

**Graphical Abstract:**
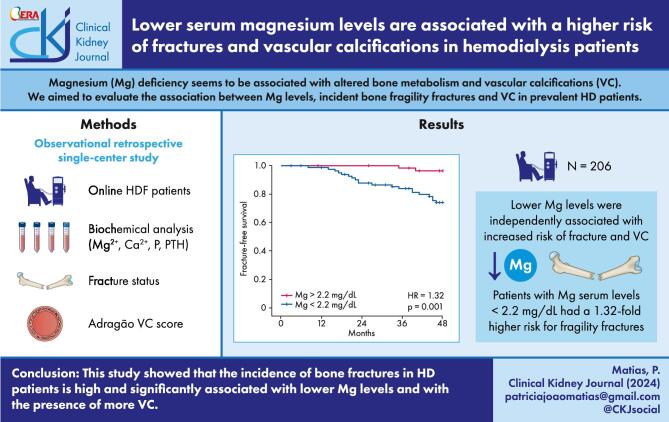

KEY LEARNING POINTS **What was known:**

- Chronic kidney disease is associated with abnormalities of bone and mineral metabolism that predispose patients to an increased risk of fragility fractures, which are an important cause of morbidity and mortality in hemodialysis (HD) patients.
- Hypomagnesemia has been associated with a higher incidence risk of fractures in the general population due to direct and indirect effects on bone metabolism.
- There is some evidence suggesting that HD patients with vascular calcifications are more likely to experience bone fractures.

**This study adds:**

- In this study, lower serum magnesium (Mg) levels and a higher vascular calcification score were found to be independent predictors of fragility fractures in HD patients.
- The fracture risk was higher in patients with intact parathyroid hormone levels <300 and >800 pg/mL. This indicates that low and high bone turnover increases bone fragility and the likelihood of bone fractures.

**Potential impact:**

- Therapy with Mg and vitamin D may be protective in preventing fractures in HD patients.

## INTRODUCTION

Bone fractures are an important cause of morbidity and mortality in hemodialysis (HD) patients [1]. Declining kidney function is associated with abnormalities of bone and mineral metabolism that predispose patients to an increased risk of fragility fractures [2]. The mineral and bone abnormalities accompanying chronic kidney disease (CKD) are collectively known as CKD–mineral and bone disorder (CKD-MBD) [2]. This condition is often associated with low bone volume and/or mineralization, and is caused by an imbalance of bone reabsorption and new bone formation [3]. Besides the classical features that are also present in the general population, features specific to CKD-MBD, such as hyperphosphatemia, diminished activation of vitamin D, secondary hyperparathyroidism and elevated fibroblast growth factor–23, alongside the exposure to medications altering bone metabolism in patients with CKD, are likely to contribute to fracture risk [4]. In the general population, lower serum magnesium (Mg) seems to be associated with a higher incidence risk of fractures [5], but there are still few studies on HD patients. Mg deficiency might affect bone directly (by reducing bone stiffness, increasing osteoclasts and decreasing osteoblasts) and indirectly (by interfering with parathyroid hormone—PTH) and vitamin D, promoting inflammation/oxidative stress and subsequent bone loss [6]. Extra-skeletal calcifications are one of the features of CKD-MBD. Vascular calcifications (VC) are the inappropriate and pathological deposition of minerals in the form of several calcium and phosphate salts into vascular tissues. VC are independent predictors of cardiovascular events and mortality, and their prevalence increases with decreasing renal function and generally follows a progressive course [7, 8]. Even though the pathogenic factors linking VC and bone fragility are not apparent, low bone volume, evaluated using bone biopsy, was a significant risk factor for VC in uremic patients [9, 10]. Schulz *et al.* [11] demonstrated that patients with the highest degree of aortic calcification had the lowest bone mineral density (BMD). In the same cohort followed for 2 years, bone loss was more significant in patients with progressive VC [11]. In agreement with these results, another study has shown that after 4 years of follow-up, subjects with the most severe aortic calcification had a lower bone mass and a higher incidence of new osteoporotic fractures [12]. However, the EMITRAL study (Mineral metabolism disorders, vertebral fractures and aortic calcifications in stable kidney transplant recipients: The role of gender) was one of the few studies that did not show any relationship between the development of vertebral fractures and VC in stable kidney transplant recipients [13]. It seems reasonable to assume that dialysis patients with evident vascular VC could be more likely to experience a fracture. This study aimed to evaluate the association between predialysis serum Mg levels, the risk of incident bone fragility fractures and the prevalence of VC in a cohort of prevalent HD patients.

## MATERIALS AND METHODS

### Study design

An observational, retrospective, single-center study was performed on a cohort of prevalent HD patients. The study was conducted in accordance with the Declaration of Helsinki and approved by the Ethics Committee of NephroCare Portugal and NOVA Medical School.

### Eligibility criteria

The inclusion criteria were age above 18 years old and the capability to give informed written consent.

#### Study population

All the patients were submitted to online hemodiafiltration with acetic acid in the dialysate, a dialysate calcium concentration of 1.25 or 1.5 mmol/L, and an Mg concentration of 1 mmol/L. The majority (97%) performed 4 h of HD three times a week. All received native vitamin D supplementation with cholecalciferol 2700 IU thrice weekly after HD during the study. The following data were analyzed: age, gender, prior kidney transplantation, presence of diabetes, hypertension, coronary artery disease, cerebrovascular disease and peripheral vascular disease. The mean body mass index (BMI) at the start of HD was evaluated. Medication with doses [active vitamin D or its analogs, phosphate binders, diuretics, proton pump inhibitors (PPIs) and darbepoetin] and daily dietary Mg were also registered. Informed consent was obtained from all subjects involved in the study.

### Fracture status

Fractures during the study's follow-up were identified through patient interviews and chart reviews. If they resulted from trauma equivalent to a fall from standing height or less, they were classified as fragility fractures [14]. High-trauma fractures and fractures of fingers, toes, face and skull were excluded. All fractures were confirmed by radiographs or radiology reports.

### Biochemical analysis

Serum calcium, phosphorus, Mg, intact PTH (iPTH), bone alkaline phosphatase (bALP), hemoglobin, albumin and C-reactive protein were recorded quarterly during the study follow-up. All blood samples were collected in midweek sessions before HD. Serum Mg was measured using a xylidyl blue colourimetric method on a Roche/Hitachi Cobas c 701 analyser (Roche Diagnostics, Mannheim, Germany). The usual range of values is 1.6–2.6 mg/dL.

### VC score

Simple VC score was evaluated through radiography of the pelvis and hands by Adragao score [15] for all patients at the beginning of HD by one of the study's researchers and confirmed by a second independent researcher. The kappa agreement value was 0.82 between the two observers. This vascular score is based on the analysis of plain radiographic films of the pelvis and hands. Pelvis films were divided into four sections by two imaginary lines: a horizontal line over the upper limit of both femoral heads and a median vertical line over the vertebral column. Hand films were divided for each hand by a horizontal line over the upper limit of the metacarpal bones. Pelvis films evaluated iliac and femoral arteries (iliofemoral score), and hand films evaluated radial and digital arteries (hand score). Any VC lining the vessel walls either in an irregular pattern or in a linear pattern was considered. The presence of VC in each section was rated 1, and its absence as 0. The final score was the sum of all sections, ranging from 0 to 8. Adragao *et al.* [15] found that a simple VC score ≥3 was associated with increased cardiovascular events and mortality.

### Statistical analysis

The incidence of fracture was expressed as the total number of fractures per 1000 patient-years of follow-up. The arithmetic means of the quarterly laboratory results obtained during follow-up were used for statistical analysis. Variables were expressed as frequencies for categorical variables, mean values with standard deviation for normally distributed variables, and median (interquartile ranges) values for non-normally distributed variables. Differences in mean values between patients with and without fractures were evaluated using the unpaired Student's *t*-test for parametric data and the Mann–Whitney U test for nonparametric data.

Cox regression was used for multivariable analysis [95% confidence interval (CI)], with time to fracture as the dependent variable and Mg as the independent variable. This analysis was adjusted for age, female gender, time on HD, diabetes, BMI, serum albumin, Mg, bALP, iPTH <300 or >800 pg/mL, therapy with active vitamin D and VC score ≥3. Survival curves were estimated using the Kaplan–Meier analysis and compared by the log-rank test. The Mg cut-off value used in survival curves was determined via a receiver operating characteristic (ROC) curve. Statistical analysis was performed with SPSS system 23.0. A *P* < .05 was considered statistically significant.

## RESULTS

The study included 206 patients who were evaluated since they started HD, with a median follow-up of 58 (4–375) months, 121 (59%) males and 85 (41%) females, with a mean age of 68.3 ± 13.1 years at the beginning of HD. Ninety-nine (48%) patients had diabetes and 74 (36%) had hypertension. Coronary artery disease was diagnosed in 64 (31%) patients, cerebrovascular disease in 49 (24%) and peripheral vascular disease in 56 (27%). The mean BMI at the start of HD was 26.8 ± 4.2 kg/m^2^. During the study, 64 (31%) patients were taking active forms of vitamin D: 25 (12%) patients under oral alfacalcidol, with a median dose of 2 (1–9) μg/week, and 39 (19%) under intravenous paricalcitol, with a median dose of 5 (2.5–15) μg/week. Twenty-seven (13%) were under calcimimetics (oral cinacalcet) with a median dose of 30 (15–90) mg/day, and four (2%) patients have been submitted to parathyroidectomy. None of the patients was taking another anti-osteoporotic treatment. In the studied period, 74 (36%) patients were under therapy with phosphate binders: 45 (22%) patients were taking calcium acetate/Mg carbonate with a median dose of 1675 (1340–6030) mg/day, 19 (9%) patients were under sevelamer with a median dose of 3200 (1600–4800) mg/day, and 10 (5%) were taking sucroferric oxyhydroxide with a median dose of 1000 (500–1500) mg/day. One hundred and thirty-two (64%) patients did not take phosphate binders.

Only 16 patients (8%) were taking diuretics. On the other hand, PPIs were very common, with 148 patients (72%) using them. Unlike other studies [16, 17], PPIs were not correlated with Mg levels.

### Incidence and characterization of fragility fractures in a prevalent HD population

There were 37 episodes of fragility fractures during the follow-up of the study (median of 58 months), corresponding to an overall incidence rate of 29 per 1000 person-years. The main localizations of fractures were: forearm 24% (*n* = 9), hip 21%, (*n* = 8), leg 19% (*n* = 7), arm 13% (*n* = 5), spine 11% (*n* = 4) and ribs 11% (*n* = 4). Three (8%) patients presented more than one fracture episode, and five (13%) patients who had a fracture during the study had already presented a fragility fracture before the beginning of HD. Prior kidney transplantation was not associated with more fractures. The median HD time to the first fracture was 42 months.

### Patients with fractures had lower Mg serum levels and more VC (univariable analysis)

Compared with patients without fractures, patients who presented episodes of fracture were more frequently female (*P* < .001), older (*P* = .002) and diabetic (*P* < .001), with lower BMI (*P* = .03), longer HD vintage (*P* < .001), and lower albumin (*P* < .001) and Mg levels (*P* < .001). These patients were also less likely to receive active vitamin D therapy (*P* = .001) and presented a higher VC score (*P* < .001). Therapy with phosphate binders, calcimimetics and PPIs did not correlate with increased fracture risk or calcium concentration in the dialysate (Table 1). Patients treated with calcium acetate/Mg carbonate showed higher Mg levels (2.7 ± 0.5 vs 2.4 ± 0.3 mg/dL, *P* < .001).

**Table 1:** Clinical and laboratory parameters in patients with and without fragility fractures (univariable analysis).

	Without fractures (*n* = 169)	With fractures (*n* = 37)	*P*
Age (years)	67.8 ± 13.1	72.1 ± 14.2	**.002**
Female gender (%)	38.3	57.1	**<.001**
HD vintage (months)	41	72	**<.001**
Prior kidney transplant (%)	9	7	NS
Diabetes mellitus (%)	41.6	54.2	**<.001**
Hypertension (%)	34.6	32.1	NS
Coronary disease (%)	33.9	35.2	NS
Peripheral arterial disease (%)	27.1	25.9	NS
Cerebrovascular disease (%)	23.8	25.1	NS
BMI (kg/m^2^)	27.1 ± 6.4	25.0 ± 3.3	**.03**
Dietary Mg intake (g/day)	0.31 ± 0.03	0.29 ± 0.05	NS
PPIs therapy (%)	72	70	NS
Phosphate binders use (%)	37.1	33.8	NS
Active vitamin D therapy (%)	29.9	18.6	**.001**
Calcimimetic therapy (%)	9.6	8.7	NS
Parathyroidectomy (%)	3.1	3.9	NS
Hemoglobin (g/dL)	11.3 ± 0.8	11.0 ± 0.6	NS
CRP (mg/dL)	0.7 ± 0.3	0.9 ± 0.2	NS
Albumin (g/dL)	3.9 ± 0.2	3.5 ± 0.3	**<.001**
Calcium (mg/dL)	8.9 ± 0.6	9.2 ± 0.5	NS
Phosphorus (mg/dL)	4.7 ± 1.1	4.4 ± 1.3	NS
Mg (mg/dL)	2.8 ± 0.4	2.3 ± 0.3	**<.001**
bALP (ug/L)	25	32	NS
iPTH (pg/mL)	372	341	NS
Vascular calcification score (0–8)	3	6	**<.001**

CRP, C-reactive protein; NS, not significant.

Forty-three patients (21%) had Mg levels <2.2 mg/dL. This group had a higher fracture incidence rate of 58 per 1000 person-years (25 episodes of fracture) compared with patients with Mg >2.2 mg/dL (*n* = 163), who had only 12 episodes of fragility fractures—an incidence rate of 19 per 1000 person-years.

Only five fracture episodes occurred in the group with iPTH between 300 and 800 pg/mL (*n* = 82)—a fracture incidence rate of 16 per 1000 person-years. However, there were 20 episodes of fracture in the group with iPTH <300 pg/mL (*n* = 104)—a fracture incidence rate of 39 per 1000 person-years. In the group with iPTH >800 pg/mL (*n* = 20), there were 12 fracture episodes—with the highest incidence rate of 77 per 1000 person-years. Patients with iPTH <300 pg/mL and >800 pg/mL presented significantly lower Mg levels compared with those with iPTH between 300 and 800 pg/mL (*P* < .001).

### Mg levels and VC were independently associated with the development of fragility fractures (multivariable analysis)

In a Cox regression analysis, time to fragility fracture was independently associated with serum Mg <2.2 mg/dL (*P* < .001), in a model adjusted to age >80 years, female gender, time on HD >48 months, diabetes mellitus, BMI, lower albumin levels, iPTH <300 or >800 pg/mL, active vitamin D therapy, and the presence of a higher VC score (≥3) (Table 2).

**Table 2:** Time to first fragility fracture—Cox regression analysis.

Dependent variable	Multivariable Cox model	Hazard ratio	95% CI	*P*
Time to fragility fracture	Serum magnesium <2.2 mg/dL	1.49	1.15–2.66	<.001
	Age >80 years	1.98	1.56–2.62	<.001
	Female gender	2.17	1.87–2.45	<.001
	HD >48 months	1.87	1.72–2.31	<.001
	Diabetes mellitus	1.23	1.09–2.77	.008
	BMI <22 kg/m^2^	1.12	0.91–2.9	.07
	Serum albumin <3.5 g/dL	1.22	1.03–1.32	.03
	iPTH <300 or >800 pg/mL	1.23	1.14–1.27	.006
	Active vitamin D therapy	0.88	0.84–0.97	.03
	VC score ≥3	1.33	1.16–1.58	<.001

### Serum Mg <2.2 mg/dL is associated with an increased risk of fragility fractures (survival analysis)

Through ROC analysis, the cut-off value of Mg to be used in the survival analysis was determined to be 2.2 mg/dL, with an area under the curve of 0.76 (95% CI 0.66–0.83), 74% sensitivity, 73% specificity, 87% negative predictive value and a positive likelihood ratio of 2.28.

**Figure 1:**
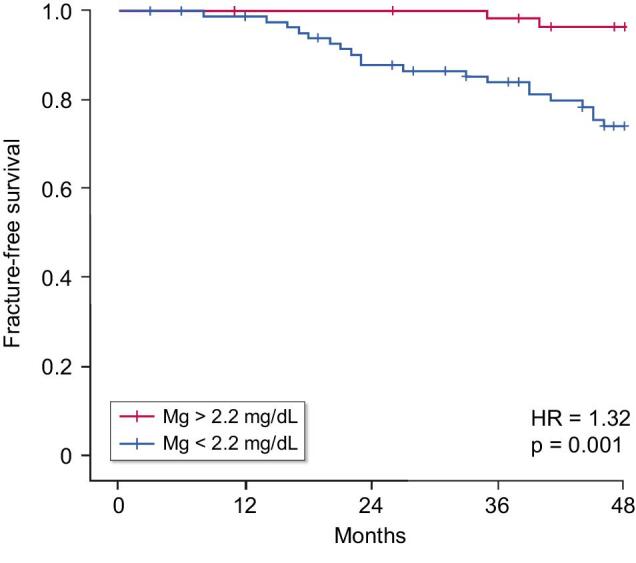
A serum magnesium (Mg) <2.2 mg/dL was associated with a significantly lower fracture-free survival (Kaplan-Meier analysis).

Kaplan–Meier fracture-free survival analysis demonstrated that patients with mean Mg serum levels <2.2 mg/dL throughout the follow-up presented a significantly higher fracture risk compared with those with Mg >2.2 mg/dL (hazard ratio 1.32; CI 1.08–4.29; *P* = .001) (Fig. 1).

## DISCUSSION

Our study found an incidence of fragility fracture in prevalent HD patients of 29 per 1000 person-years, which is approximately three times greater than that reported for elderly patients without CKD [18] Our incidence rate is similar to that presented in the Dialysis Outcomes and Practice Patterns Study (DOPPS) [1], and the US Renal Data System [19]; however, in this study, we analyzed all radiologically proven fractures, whether the patients were hospitalized or not, which may have overestimated the fracture incidence rate.

Incident vertebral fractures were identified in 11% of the patients of our HD cohort, compared with a prevalence of 55.3% in a study of 387 patients by Fusaro *et al*. [20]. The authors in that study determined the prevalence of historical vertebral fractures in HD patients, irrespective of symptoms, radiologically identified using a specialized, quantitative vertebral morphology software (MorphoX-Press). In contrast, we only identified the incidence of new symptomatic fractures. Demographic risk factors, as shown in other studies [21], including female gender, older age, diabetes, low BMI, longer dialysis vintage and a previous history of fracture, were also highly associated with an increased risk of fragility fractures. Prior kidney transplantation was not associated with a higher risk of fractures, probably due to the reduced number of events and the absence of data on corticosteroid exposure. A low BMI in CKD patients often indicates malnutrition and sarcopenia (loss of muscle mass), which can lead to frailty and increased susceptibility to falls and fractures [21]. Patients with low BMI are also more likely to have low BMD, a significant risk factor for fractures. The reduced mechanical load on bones from lower body weight can also contribute to decreased bone strength [21].

Our data are also consistent with previous reports showing an association between the risk of fractures in the HD population and lower serum albumin levels [22] and no significant fracture association with serum calcium and phosphorus [23]. Patients treated with calcium acetate/Mg carbonate presented higher Mg levels. However, no significant impact in fractures was observed in this group of patients, possibly due to the few events recorded. A recent study, however, showed an association between the use of Mg-based antiacids in both nondialysis CKD patients and long-term dialysis patients and an increased risk of hip fracture [24]. The authors concluded that excess of Mg can negatively impact bone mineral density and strength. CKD patients often have altered mineral metabolism, including abnormalities in Mg levels, which can exacerbate bone fragility.

On the contrary, like in other studies, we found that lower predialysis Mg serum concentrations were associated with a higher incidence of fragility fractures [6, 25]. The adult human body contains ∼24 g of Mg, mostly (99%) in the bone, muscles and soft tissues. Serum Mg concentration does not correlate with tissue pools except interstitial fluid and bone. Only 1% of total body Mg is present in extracellular fluids. Only 0.3% of total body Mg is found in serum, so serum Mg concentrations are poor predictors of intracellular/total body Mg content [26] compared with serum ionized free and total Mg which are not performed in daily routine but only for investigation. We used a xylidyl blue colourimetric method to access Mg serum levels, although atomic absorption spectrophotometry was more accurate.

In blood, 20%–30% of Mg is bound to albumin and other serum proteins, with around 60% free or bound to phosphate, citrate and other negatively charged solutes. The free and bound serum Mg amount varies with pH and azotemic solutes, and the free fraction is reduced in CKD patients [26].

As for serum phosphate levels, there is a natural intra-individual circadian variation (10%–15%) in Mg serum levels. Mg levels are more stable and reach a steadier state during the morning hours (6–10 a.m.) [26]. In our study, the Mg was measured at different hours of the day before the HD session, although several determinations were performed that could overcome this fact.

Although mild hypermagnesemia may be preferable for HD patients in terms of survival, there are some concerns about its potential harmfulness to bone metabolism. While Mg is an essential bone mineral, excess Mg can disturb bone mineralization [6]. According to the Japanese Society for Dialysis Therapy—Renal Data Registry however, mild hypermagnesemia was not associated with an increased risk of hip fracture in HD patients [27]. Lower serum Mg levels were significantly associated with an increased risk of hip fracture. Notably, the population-attributable fraction of serum Mg levels for incident hip fracture was higher than that of serum calcium, serum phosphate and iPTH levels, suggesting that low Mg may contribute to the fracture burden to a greater degree than other factors related to mineral and bone disorder in CKD. In another longitudinal study on HD patients, those with lower Mg and lower BMD had a 9.21-fold higher risk of fractures when compared with patients with higher serum Mg concentrations and high BMD. Therefore, adding Mg levels and lumbar spine BMD significantly improved the prediction of fractures [28].

Mg toxicity is relatively rare in dialysis patients but can occur, mainly if there is an accumulation due to excessive intake or insufficient dialysis clearance. Mild toxicity that can cause nausea, vomiting, hypotension and lethargy only happens with Mg levels >2.6–4 mg/dL. In dialysis patients, the ionized Mg fraction—the biologically active form—can be reduced due to various factors, including changes in protein binding and acidosis [29].

Many vitamin D–responsive genes are expressed in bone-forming osteoblast cells and bone-reabsorbing osteoclast cells, including the tumor necrosis factor ligand family gene of the receptor activator of nuclear factor-κB ligand (RANKL), which is involved in osteoclastogenesis, and is modulated by the 1,25-dihydroxyvitamin D [30]. In osteoporosis patients, there is a higher rate of Mg deficiency in those with vitamin D deficiency compared with those with normal levels of vitamin D [31]. Interestingly, a study conducted among osteoporotic patients showed much higher prevalence rates of Mg deficiency or insufficiency among people with insufficient 25-hydroxyvitamin D [25(OH)D] than among those with sufficient 25(OH)D serum levels [25].

Several studies have shown that Mg inhibits VC [32, 33]. Mg may counteract VC by inhibiting intestinal phosphate uptake as a result of phosphate binding, by systemic effects on both promoting and inhibiting factors of calcification or by local effects at the vascular tissue level [33]. Mg also counterbalances calcification of vascular smooth muscle cells (VSMCs) and aortic tissue *in vitro* and inhibits expression of osteogenic transcription factors, including runt-related transcription factor 2 (RUNX 2), osterix and bone morphogenetic protein-2 (BMP-2); and genes associated with matrix mineralization, including bALP [34–36]. The local inhibiting effects of Mg on VC may be mediated in multiple ways. First, Mg passively interferes with the maturation of amorphous calcium/phosphate particles, thereby preventing the formation of stable hydroxyapatite [37]. Second, Mg inhibits calcium influx via L-type calcium channels in VSMCs, which affects vascular tone [38]. It is plausible that inhibition of calcium influx channels limits the intracellular rise of calcium concentration associated with VC, which may inhibit possible stimulating effects of intracellular calcium on the calcification process [39]. Third, Mg acts on the calcium-sensing receptor (CaSR) expressed in VSMCs. This expression is decreased in calcifying conditions, and stimulation of the CaSR by calcimimetics inhibits VSMCs calcification [40].

*In vitro*, studies also demonstrated that Mg supplementation reduces phosphate-induced calcification of VSMCs through the inhibition of osteogenic transdifferentiation by the Wnt/β-catenin signalling pathway [41]. Mg deficiency may accelerate VC and atherosclerosis, thus causing cardiovascular disease [42]. In uremic rats, Mg supplementation prevents aortic calcification and improves mineral metabolism and renal function [41]. Theoretically, Mg may help prevent the progression of coronary artery calcification, which predicts cardiovascular events and mortality among CKD patients. In several epidemiological studies, circulating Mg is inversely associated with coronary artery calcifications [42]. A randomized controlled trial showed that oral Mg oxide delayed coronary artery calcification progression in CKD stage 3–4 patients [32].

An animal model study showed a potential interaction between vitamin D and Mg [43]. This study revealed that combining Mg with calcitriol treatment can reduce hypercalcemia and similarly suppress PTH while protecting, at least in part, the vasculature from calcium and phosphate deposition. These results demonstrate that calcitriol can increase VC under certain circumstances, an effect that is attenuated in the presence of increased Mg. Notably, the calcitriol-induced reduction in vascular TRPM7 protein expression was partially abrogated by Mg co-treatment [43]. These data suggest that the benefit of this combined treatment likely involves preventing reductions in TRPM7 expression and increasing the relative entry and availability of Mg (reducing calcium/Mg ratio) in the VC-susceptible microenvironment.

Bone metabolism disorders and the tendency to develop vascular VC in CKD patients seem to be directly connected. Reduced bone formation has been associated with coronary calcifications in CKD patients not yet on dialysis [44]. Some studies suggest a link between low BMD, arterial calcifications, and vascular stiffness in dialysis patients [45, 46]. Our results also showed that prevalent HD patients with a higher VC score presented an increased risk of fractures.

Like in the general population, older CKD patients are also prone to develop osteoporosis. The features of osteoporosis in CKD patients are low trabecular bone volume and disrupted micro-architecture, even without significant abnormalities in mineralization and bone turnover [47]. Bone loss is mainly from the cortical bone in subjects with CKD-MBD, and their iPTH, bALP, klotho, sclerostin and fetuin-A levels are pronouncedly altered [48]. Due to the high prevalence of CKD-MBD and osteoporosis in CKD subjects, both conditions commonly exist simultaneously. However, CKD-MBD is more complex than osteoporosis, and it influences bone quality, contributes to high rates of fracture and, most importantly, may facilitate the appearance of VC in CKD patients. Although both result in bone fragility, they have different pathophysiological mechanisms to destroy the bone. Osteoporosis is induced by excessive osteoclastic bone resorption, insufficient osteoblastic activity and deficient bone formation in postmenopausal women and older subjects. However, CKD-MBD is related to altered mineral metabolism and the imbalance of pro- and anti-calcification factors, which induce either high or low-turnover bone disease in CKD patients [2].

Our study demonstrated that fracture risk was higher in patients with iPTH <300 and iPTH >800 pg/mL. It is known that the level of PTH in CKD patients may suggest the histologic change associated with bone fracture. When serum PTH level is <150 pg/mL, the fracture is most likely due to adynamic bone disease or osteomalacia. However, in patients with higher PTH (> 600 pg/mL), the leading cause of fractures is most likely due to osteitis fibrosa, which is prone to developing fractures despite frequently increased trabecular bone mass [49]. Unfortunately, we lack evidence of an association between fracture incidence and the histologic type of bone disease. Despite definitive proof, low and high bone turnover increases the fracture risk as both increase bone fragility. This might be partly explained by the absence of analysis of cortical bone, which is predominantly affected in CKD [50]. Besides bone biopsies, new bone image techniques, such as high-resolution computed bone tomography, may contribute to understanding the role of cortical on the risk of fragility fractures in CKD patients.

Alkaline phosphatase (ALP) is mainly a biochemical marker of bone turnover, and it is usually used to monitor metabolic bone disease, particularly the management of CKD-MBD. A higher ALP might be associated with fracture through high bone turnover [51]. Indeed, Park *et al.* [52] found that serum ALP was negatively associated with BMD assessed by dual-energy X-ray absorptiometry (DEXA) in HD patients. However, ALP is primarily secreted by the liver and bone, and the intestine, kidneys and leukocytes also secrete a small amount. Thus, bALP monitoring is preferred in assessing bone mineral metabolism, particularly bone formation [53]. In a single-center cohort study, Iimori *et al.* [54] investigated 485 HD patients with a median follow-up time of 39.9 months. They found that serum bALP was associated with any type of incident fracture. In our cohort of patients, bALP did not show any association with the presence of fragility fractures.

A possible protective role of active vitamin D in preventing fractures in HD patients is also shown in this study. Active vitamin D administration is associated with increased bone strength in animal models [55], and a prospective, randomized, placebo-controlled, double-blind study showed improved BMD assessed by DEXA with lowering plasma PTH levels [56]. Poor performance on neuromuscular function tests also may identify those at higher risk of fracture in CKD. This is likely to reflect, to some extent, their higher risk of falls due to impaired muscle strength. The mechanism underlying this association is unclear, but a reduction in active vitamin D seems important [57, 58]. In non-CKD populations, pooled data from 11 double-blind, randomized, controlled trials of oral native vitamin D supplementation demonstrated an association, although not significant, of vitamin D (≥800 IU daily) and reduced hip fracture and any nonvertebral fracture in participants with 65 years of age or older [59]. Nevertheless, we still have insufficient evidence in CKD patients correlating the correction of serum 25(OH)D levels with reduced fracture risk [48, 60].

This study regarding the incidence of bone fragility fractures and its risk factors, namely Mg and the presence of VC, in a large HD population, with an extended follow-up, had some limitations. First, this study's retrospective and observational nature precludes the conclusions of a causal relationship. Also, the relatively low number of events could lead to potential statistical bias, and we can not exclude that some variables could act as confounders. Second, VC were evaluated by the Adragao score, which is not a gold standard method for assessing VC, compared with a better performance shown with the Kaupilla score (lateral abdominal X-ray) or computed tomography. Third, we did not evaluate the participants’ BMD. However, the ability of BMD, as measured by DEXA in dialysis patients, to predict the risk of fractures is still dubious. Fourth, this was a single-center study, so the results cannot be generalized. However, all the participants were treated by the same physicians and underwent uniform laboratory measurements during a long observation period, which might guarantee the accuracy of our results.

## CONCLUSIONS

The incidence of bone fragility fractures in HD patients is high, and its risk increases with age, female gender, low serum albumin, lower Mg levels and the presence of more VC. Prevalent HD patients with low or high iPTH levels had a higher fracture risk. Active vitamin D therapy seemed to have a protective role. Patients with mean Mg <2.2 mg/dL showed an increased risk of fragility fractures during the follow-up of the study.

In patients with CKD, the administration of Mg can significantly slow down the progression of vascular disease. Most evidence supports the beneficial effects of VC. Mg supplements also influence mineral bone metabolism by improving serum calcium and PTH levels. Current clinical evidence shows that administering Mg to CKD 1–5 patients is safe, without concerns for severe hypermagnesemia or harmful interference in CKD-MBD. Further studies focused on long-term clinically relevant outcomes are still necessary.

## Data Availability

The article's data will be shared upon reasonable request to the corresponding author.
